# Development and validation of fall risk perception scale for patients with Parkinson’s disease

**DOI:** 10.3389/fpsyg.2024.1289067

**Published:** 2024-02-28

**Authors:** Xin Yang, Meiqi Yao, Zhiting Guo, Xuhui Shen, Jingfen Jin

**Affiliations:** ^1^Nursing Department, The Second Affiliated Hospital Zhejiang University School of Medicine (SAHZU), Hangzhou, China; ^2^Faculty of Nursing, Zhejiang University School of Medicine (SAHZU), Hangzhou, China; ^3^Faculty of Nursing, Huzhou Teachers College, Huzhou, China

**Keywords:** Parkinson’s disease, risk perception, fall, scale development, reliability, validity, safety nursing

## Abstract

**Background:**

Perception assessment plays an important role in fall risk awareness and fall prevention. Parkinson’s disease patients with motor dysfunction are at high risk of falling. Currently, no instrument has been explicitly crafted to assess the risk perception of fall in PD patients. The purpose of this study was to develop and validate the fall risk perception scale for PD patients (FRPS-PD), providing healthcare professionals with a effective assessment tool to enhance proactive fall prevention initiatives.

**Method:**

Based on the Proactive Health theory and Risk Perception Attitude (RPA) Framework, the questionnaire was developed through literature review, semi-structure interview, expert consultation and pilot testing. A total of 428 patients with PD from Grade A tertiary hospitals in Shanghai, Hangzhou and Anhui from January 2023 to July 2023 were recruited. The items and dimensions in the scale were explored and confirmed using item-analysis, content validity, exploratory factor analytical (EFA), confirmatory factor analytical (CFA), internal consistency and test–retest reliability analysis.

**Results:**

A total of 16-items, 2-dimensions structure were identified, including 12 items of risk perception and 4 items of self-efficacy dimension. The cumulative variance of EFA model was 73.669%, further CFA showed that acceptable model fit (χ^2^/df = 2.226, RMSEA = 0.074, NF = 0.928, TLI = 0.951, CFI = 0.959, GFI = 0.887 and AGFI = 0.848). The content validity index was 0.956. The reliability of the scale was 0.952 using Cronbach’s α coefficient method. The test–retest reliability was 0.944.

**Conclusion:**

The FRPS-PD is a valid and reliable measurement for evaluating fall risk perception level for individuals with PD in mainland China.

## 1 Introduction

Parkinson’s Disease (PD) is characterized by the dopaminergic neuronal loss in specific areas of the substantia nigra and widespread intracellular protein (α-synuclein) aggregations (Poewe et al., 2017; Armstrong and Okun, 2020). These progressive neurodegenerative disorder causes a range of clinical features, including motor and nonmotor symptoms (Reichmann, 2017; Armstrong and Okun, 2020). Motor impairment include bradykinesia, tremor, rigidity and gait disturbance (Armstrong and Okun, 2020), as well as non-motor symptoms consist of hyposmia, impaired color vision, hallucinations, pain, anxiety, depression, sleep disturbance and bladder hyper-reflexia (Schapira et al., 2017; Armstrong and Okun, 2020).

The incidence and prevalence of PD increases steadily with age (Pringsheim et al., 2014). Research conducted in China indicates that the prevalence rate of PD is 1.37% among individuals aged 60 and above, while in those aged 65 and older, the rate rises to 1.63% (Qi et al., 2021). PD patients exhibit a heightened susceptibility to falls, primarily attributed to gait-related indices and abnormal posture with knee flexion (Pallavi et al., 2022), with an estimate that 35 to 90% experience at least one fall annually and 39% (range 18 to 50%) reporting recurrent falls (Allen et al., 2013). These falls not only result in severe secondary complications such as hemorrhage, fractures, and pain but also increase the risk of mortality (Liu et al., 2022). Moreover, fall exacerbate both physical and psychological stress experienced by patients, consequently their ability to care for themselves and leading to a decline in their overall quality of life (Rahman et al., 2008; Soh et al., 2013). The subsequent medical costs associated with hospitalizations due to falls impose a considerable financial burden on both the patients, their families and the whole society (GBD 2016 Parkinson's Disease Collaborators, 2018; Macchi et al., 2020).

Perception is a core concept grounded in the Proactive Health theory (Song et al., 2023; Wang et al., 2023) and the Risk Perception Attitude (RPA) Framework (Rimal, 2003; Rimal et al., 2009). This concept emphasizes the importance of leveraging an individual’s distinctive advantages in health management. It advocates for proactive information acquisition to promote health and prevent diseases, shifting the focus from reactive medical interventions to an active, self-motivated approach toward health maintenance (Zhang et al., 2022). Fall risk perception refers to an individual’s judgment of their own imminent risk of falling, based on a subjective evaluation of environmental risk factors and their own gait ability, which in turn informs preventive behaviors against falls (Choi et al., 2021; Dabkowski et al., 2022). From the perspective of Proactive Health theory, patients can recognize the risk of falls and proactively develop an awareness of prevention, then such proactive behavior serves as an intrinsic motivator for effective fall management and care. However, various factors including education level, age and medication use (Souza et al., 2022) can influence the consistency between perceived and objective fall risk, which is called “perceived bias” (Bao et al., 2022). Perception bias has been noted that both underperceived and overperceived the risk of falls can be detrimental (Aycock et al., 2019). People who overperceived their risk of falling often tend to hold excessive fear and consequently diminish their physical activities (Huang et al., 2022), resulting in adverse outcomes including muscle atrophy, sarcopenia, and frailty, which ultimately impede long-term fall prevention efforts (Tymkew et al., 2023). Conversely, individuals who hold insufficient perception toward fall risk would neglect established fall-prevention guidelines and are prone to engage in high-risk behaviors, thereby increasing their risk of falls ultimately (Yardley et al., 2006). A study by Lim et al. (2018) revealed that among hospitalized patients aged 65 and above in Singapore, only 31.3% accurately perceived their fall risk, while 50.7% overperceived and 18% underperceived it. In a similar vein, research by Bao et al. (2022) on Chinese hospitalized patients aged over 18 found that 27.5% underperceived and 10.6% overperceived their fall risk. It has been established that individuals with perception biases are at a heightened risk of recurrent falls (Gálvez-Barrón et al., 2013), while accurate perception has been shown to reduce the incidence of falls by up to 50% (Christiansen et al., 2020). Therefore, the integration of perceived risk into existing fall assessment protocols for individuals with PD may significantly enhance clinical fall management, thereby reducing the incidence of falls.

Currently, fall risk perception questionnaires have been developed for diverse populations. A scale formulated by Bao et al. (2023) aimed at assessing the perception of fall direction among elderly individuals in the community. Similarly, South Korean researchers (Choi et al., 2021) designed the Fall Risk Perception Questionnaire for hospitalized Patients (FRPQ), which contains three primary dimensions: environmental factors, personal activity, and physical condition. However, the unique clinical symptom and signs of PD differentiate fall risk perception from that general hospitalized patients and older community adults, consequently, the existing fall risk perception scales fail to encapsulate the specific characteristics of risk perception in PD patients, resulting in imprecise evaluations. At present, there is no instrument designed explicitly to assess the perceived risk of fall in PD patients. To address this issue, we developed the fall risk perception scale for PD patients (FRPS-PD), and established its reliability and validity for individuals with PD. This scale would be helpful to promote early identification of fall risk misperception, and provide healthcare professionals with personalized fall prevention education and intervention strategies, ultimately reducing the incidence of falls.

## 2 Materials and methods

### 2.1 Study design

This study was conducted to develop and validate the FRPS-PD in PD patients. The study protocol was approved by the Ethics Committee of the Second Affiliated Hospital of Zhejiang University School of Medicine (Research-2022-1064).

### 2.2 Development of FRPS-PD

**Figure 1:**
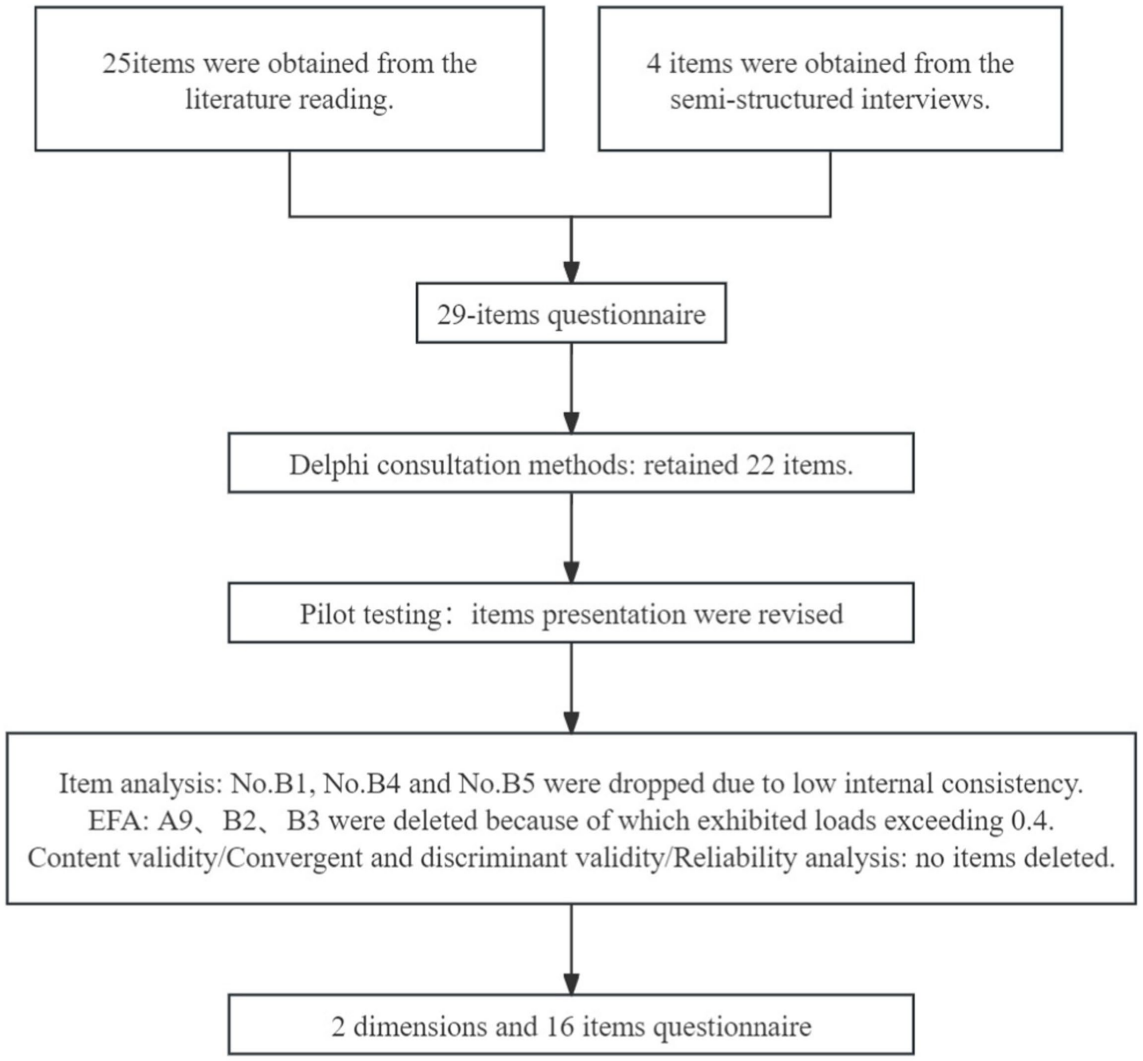
The number of items at each stage of measurement development.

The phase of scale development was guided by literature review, expert panel consultation and patient focus group cognitive interview. The number of items was decreased at each stage of measurement development, and the details were showed in Figure 1.

#### 2.2.1 Theoretical framework

According to the foundational concept of Proactive Health theory (Song et al., 2023; Wang et al., 2023), “Proactive health” is characterized by one’s voluntary actions toward health promotion, driven by intrinsic motivation. When applied to PD patients’ self-assessment of fall risk, the paradigm shifts from “passive fall prevention” to “active participation.” This approach encourages PD patients to acquire fall prevention knowledge and engage in preventive measures. The RPA framework (Rimal, 2003; Rimal et al., 2009) comprises two principal components: risk perception and self-efficacy, each acting as a vital motivator for individuals to adopt protective behaviors. Risk perception is an individual’s empirical insight, shaped by subjectively evaluating environmental risk information, whereas self-efficacy represents an individual’s internal assessment of their ability to reduce risk through protective actions. Based on RPA, incorporating the cultural background of China, this study identifies risk perception and self-efficacy as the foundational dimensions of the scale.

#### 2.2.2 Items generation

A mixed methods approach was used for items generation. First, Web of Science, PubMed, Science Direct, Embase, Cochrane Library databases, CNKI, Wanfang database, China Biomedical Literature Database were searched to obtain relevant literatures by searching theme and keywords such as “Parkinson disease” “fall” and “risk perception” with no limits on the year of publication (De Vet et al., 2005). A total of 1,795 relevant articles in Chinese and 1,765 in English were obtained and analyzed to extract initial items. After discussion by the research team, 25 items and 2 hypothesized domains were formed. The two domains consisted of 14 and 11 items, respectively. Secondly, semi-structured interviews (Kallio et al., 2016) were conducted with 10 PD patients to obtain more comprehensive understanding into their risk perception and self-efficacy toward fall. The outline of interview was presented in Supplementary Table S1. The interview content was summarized, and four additional items were added, including “I’m at risk of falling when I have hallucinations,” “I’m at risk of falling when I go over obstacles,” “I’m at risk of falling when I picked up speed suddenly,” and “I am willing to use a walker to prevent falling if required.” Finally, these 29 items formed the original version of FRPS-PD (Supplementary Table S2).

#### 2.2.3 Items tailoring and modification

A panel of 28 experts specializing in neurology medicine, geriatric medicine, clinical nursing, nursing management, and nursing education evaluated each item using Delphi consultation methods (Mcpherson et al., 2018) in December 2022. The average age of experts was (47.18 ± 5.89) years, working experience was (26.50 ± 7.53) years. The expert positive coefficient, expert authority coefficient and Kendall coordination coefficient were 100%, 0.898, 0.150, and 0.142, respectively (*p* < 0.001). The items were modified according to the expert opinions. In the first round of expert consultation, 7 items (No. A1, No. A4, No. A7, No. A16, No. A17, No. B1, and No. B8) were deleted, 2 items (No. A2 and No. B9) were merged, 9 items (No. A3, No. A8, No. B2, No. B3, No. B4, No. B7, No. B10, No. B11, and No. B12) were modified and 2 items were added. In the second round of expert consultation, 1 item (No. B5) was deleted, two items (No. B9 and No. B12) were modified. Finally, a pretest version of FRPS-PD with 2 hypothesized domains containing 22-item scale was formed (Supplementary Table S3).

#### 2.2.4 Pilot testing

The operability and legibility of the pretest version of FRPS-PD was confirmed through patient cognitive interview. Convenience sampling was employed to recruit 20 PD patients from the Department of Neurology of a Grade A tertiary hospital in Hangzhou during January 2023. The purpose was elucidated by the researcher, and the questionnaire was filled by participants one by one. Patient responses during the scale completion process were observed, and their experience of filling for wording or sentence were queried. The time for filling a questionnaire was recorded. Since no major suggestions, only the content of items was fine-tuned based on their feedback.

### 2.3 Validation of FRPS-PD

Based on the COSMIN (Consensus-based Standards for the Selection of Health Status Measurement Instruments) checklist guidelines (Prinsen et al., 2018; Terwee et al., 2018), the psychometric properties of the FRPS-PD were evaluated, including item analysis, structural validity, convergent and discriminant validity, reliability, floor and ceiling effects, and criterion validity.

#### 2.3.1 Participants and data collection procedure

A cross-sectional survey was performed. Patients with PD from grade A tertiary hospitals in Shanghai, Hangzhou and Anhui were recruited via the convenience sample method from January 2023 to July 2023. The following criteria were employed for inclusion: (a) diagnosed Parkinson’s Disease by 2015 International diagnostic criteria (Postuma et al., 2015); (b) aged of 18 years older; (c) adequate self-care ability with ADL (activities of daily living scale) > 60 score (Lawton and Brody, 1969); (d) absence of cognitive dysfunction with MMSE (Mini-mental State Examination) > 27 score (Folstein et al., 1975); (e) being able to read and articulate in Mandarin; (e) agreed to participate in this study. Patients with severe vestibular dysfunction, visual impairment and other conditions compromising walking and balance, with serious organic diseases and substance abuse, or with communication barriers (deafness or blindness) were excluded.

Paper based questionnaires were distributed by proficient nurses at each study site, and these questionnaires were promptly collected upon completion to ensure their completeness. Adhering to established methodological recommendations (Wu, 2010), the application of exploratory factor analysis (EFA) requires a minimum of 10 cases per item, while constructing a confirmatory factor analysis (CFA) model necessitates a sample size of at least 200. Consequently, it was determined that a minimum sample size of 420 was essential for this study. A total of 428 patients with PD enrolled in our study. The sample was divided into two subgroups according to the order of questionnaire collection to perform EFA (*n* = 200) and CFA (*n* = 228).

#### 2.3.2 Measures

##### 2.3.2.1 Demographic characteristic

A self-compiled questionnaire was applied to collect demographic information including gender, age, marital status, occupation, monthly income, educational level, medical history, body mass index, smoking, drinking, activity status, number of falls last year, medical payment, mode of living, PD duration, Hoehn-Yahr phase, PD motor classification, family history of PD, the use of beta blockers, angiotensin-converting enzyme inhibitors (ACEI) and calcium channel blockers, comorbidities, the severity of symptoms based on The Movement Disorder Society Unified Parkinson’s Disease Rating Scale-part III (MDS-UPDRS-III) (Goetz et al., 2008), and levodopa equivalent dose.

##### 2.3.2.2 Fall risk perception scale for PD patients

The FRPS-PD is a self-reported instrument designed to assess the risk perception level of fall in PD patients. As mentioned previously, the FRPS-PD contains 22 items with 2 hypothesized dimensions, namely the risk perception dimension (13 items) and the self-efficacy dimension (9 items). The item was scored on a 5-point Likert scale from 1 (strongly disagree) to 5 (strongly agree) as recommended in previous study (Ren et al., 2022). The total score is calculated by adding up the coded values, and the higher score represents higher perceived fall risk.

##### 2.3.2.3 Morse fall scale

The MFS was adopted widely to evaluate the actual risk of falls (Morse et al., 1989). MFS comprises six variables: a history of falls within a three-month period, secondary medical diagnosis, use of ambulatory aids, intravenous therapy, gait and mental status. Each item was scored, with a total score ranging from 0 to 125. Higher scores indicated an increased risk of falls. Individuals with a score less than 45 points were considered to have a low risk of falling, whereas those with a score more than 45 were considered to be at high risk (Chow et al., 2007). The MFS had great reliability (Cronbach’s α coefficient = 0.96), acceptable sensitivity (0.78) and specificity (0.83) (Morse et al., 1989). In our study, MFS was assessed by nurses in the ward.

#### 2.3.3 Data analysis

The data analysis was performed utilizing SPSS V.25.0 and AMOS V.28.0. Categorical data were presented in terms of frequencies and percentages, while continuous data were summarized by means and standard deviations (SDs). Chi-square test were conducted to compare difference in demographics between two samples (EFA sample and CFA sample). *p*-values < 0.05 were considered statistically significant.

##### 2.3.3.1 Content validity

The content validity index (CVI) of the scale was assessed at both the content item level (I-CVI) and the scale level (S-CVI) based on the ratings of experts on this questionnaire. The I-CVI represents the proportion of content experts who assigned a rating of either 3 or 4 on a 4-point scale, while the S-CVI is calculated as the average of the total I-CVI scores for the scale. In accordance with the recommendations of Polit and Beck (2009), a minimum I-CVI of 0.78 and S-CVI of 0.80 are considered appropriate when the number of experts exceeds 6.

##### 2.3.3.2 Item analysis

The item analysis was conducted using the following procedures: (1) The Critical Ratio (CR) was utilized to identify items with CR values less than 3 and *p*-values greater than 0.05, which were subsequently excluded (Wu, 2010). (2) Correlation coefficient method: an item was retained when its score was significantly correlated with the total score of the scale or an item-total correlation value ranging from 0.40 to 0.80 with Pearson correlation method (Walker, 2010; Wu, 2010; Pan et al., 2023). (3) Cronbach’s α coefficient method was utilized to assess internal consistency. If Cronbach’s α was greatly increased when an item was deleted, it suggested that the item hampered the table’s internal consistency and was therefore deleted (Wu, 2010).

##### 2.3.3.3 Structure validity

EFA was first conducted to establish the structural validity of FRPS-PD, then the results were verified by CFA. Principal component analysis was employed to conduct EFA in order to ascertain the factor structure of the scale. The factorability of dataset was confirmed through an examination of the correlation matrix, the Kaiser-Meyer-Olkin Measure of Sampling Adequacy (KMO) and Bartlett’s Test of Sphericity (Browne, 2001). The correlation matrix revealed that all correlations > 0.30 (Wu, 2010), KMO ≥ 0.60 (Kaiser, 1974), and Bartlett’s Test of Sphericity was significant (*p* < 0.001) (Bartlett, 1954), thus indicating a dataset suitable for factor analysis. The criterion for factor extraction and item retention was eigenvalues >1.0, factor loading >0.45 (Wu, 2010). The factor structure was determined by examining the scree plot. For CFA analysis, the maximum likelihood method was used to explore the dimensionality of items in more detail. The goodness-of-fit of the CFA model was assessed using the Chi-square value of freedom ratio (χ^2^/df), root mean square error approximation (RMSEA), Tucker-Lewis index (TLI), comparative fix index (CFI) and goodness of fit index (GFI) (Woon, 2008). A CFA model is considered good fit when 1 < χ^2^/df < 3, and TLI, CFI, and GFI > 0.90, RMSEA < 0.08 (Woon, 2008).

##### 2.3.3.4 Convergent and discriminant validity

Average Variance Extracted (AVE) was calculated to evaluate the internal convergent validity of a factor. AVE > 0.50 implies good aggregate validity, and the values between 0.36 and 0.5 are deemed acceptable (Fornell and Larcker, 1981). Discriminative validity ensures that there is no dual loading or cross-loading of items within the scale. The model exhibits good discriminative validity when the square root of the AVE exceeds the correlation coefficient between the factor and other factors (Fornell and Larcker, 1981).

##### 2.3.3.5 Reliability

The reliability of the scale was assessed through assessment of internal consistency and stability. As the suggestion by Peters (2014), internal consistency was tested via Cronbach’s α. The intraclass correlation coefficient (ICC) was calculated by the test–retest reliability method to evaluate the stability of the FRPS-PD (Perinetti, 2018). Thirty PD patients were conveniently selected to fill in the scale again at an interval of 2 weeks. A split-half reliability value >0.70 means good reliability for Cronbach’s α, and ICC coefficient (Lance et al., 2006; Wu, 2010; Tavakol and Dennick, 2011).

##### 2.3.3.6 Floor/ceiling effect

The floor/ceiling effect for the FRPS-PD was analyzed to assess the interpretability, which be calculated as the percentage of participants score at the bottom and top of a scale. As suggested by COSMIN consensus (Mokkink et al., 2010), fewer than 15% of responses with either the lowest (score = 1) or the highest score (score = 5) were deemed acceptable, indicating negligible floor and ceiling effects.

##### 2.3.3.7 Criterion validity

The Spearman rank correlations were analyzed between the FRPS-PD and The Morse Fall Scale (MFS) to evaluate the concurrent validity and predictive validity. The correlation of |*r*| = 0.10–0.30, |*r*| = 0.31–0.60, and |*r*| = 0.61–1.00 were considered low, moderate and high, respectively (Andresen, 2000).

## 3 Results

### 3.1 Sample characteristics

A total of 428 PD patients completed the survey effectively. The age of participants ranged from 25 to 85 years (mean = 66.38, SD = 9.28). PD patients who were diagnosed less than 5 years, with missed type of motor symptom, and mild severity of PD counted the most. The detailed characteristics of participants are shown in Table 1.

**Table 1:** Characteristics of the enrolled participants (*n* = 428).

Variables	EFA (*n* = 200)		CFA (*n* = 228)		χ^2^	*p*
	Fre.	Percent (%)	Fre.	Percent (%)		
**Gender**					0.322	0.570
Male	109	54.50	118	51.80		
Female	91	45.50	110	48.20		
**Age, years**					0.609	0.435
18 ~ 40	1	0.50	2	0.88		
40 ~ 60	51	25.50	65	28.51		
>60	148	74.00	161	70.61		
**Marital status**					1.372	0.241
Single	4	2.00	9	3.95		
Married	196	98.00	219	96.05		
**Occupation**					0.215	0.643
Employed	53	26.50	65	28.51		
Retire	147	73.50	163	71.49		
**Monthly income, RMB**					3.162	0.075
<3,000	53	26.50	65	28.51		
≥3,000	147	73.50	163	71.49		
**Educational level**					0.327	0.849
Primary and below	82	41.00	96	42.11		
Middle school	102	51.00	111	48.68		
College and above	16	8.00	21	9.21		
**Medical history**					1.286	0.732
Hypertension	58	29.00	81	35.53		
Heart disease	11	5.50	10	4.39		
Diabetes	14	7.00	16	7.02		
Cerebrovascular disease	11	5.50	11	4.82		
**Body mass index, kg/m** ^**2**^					1.259	0.739
Low (<18.5)	4	2.00	7	3.07		
Normal (18.5 ~ 23.9)	137	68.50	146	64.04		
Overweight (24.0 ~ 27.9)	55	27.50	69	30.26		
Obesity (≥28.0)	4	2.00	6	2.63		
**Current smoking**	29	14.50	27	11.84	0.662	0.416
**Current drinking**	21	10.50	27	11.84	0.193	0.661
**Activity status**					0.001	0.977
Walking unassisted	173	86.50	197	86.40		
Walking aid	27	13.50	31	13.60		
**Number of falls last year**					0.360	0.835
0	157	78.50	182	79.82		
1–10	38	19.00	39	17.11		
>10	5	2.50	7	3.07		
**Medical payment**					0.609	0.435
Medical insurance	181	90.50	201	88.16		
Self-paying	19	9.50	27	11.84		
**Mode of living**					1.972	0.160
Live alone	8	4.00	4	1.75		
Live with others	192	96.00	224	98.25		
**PD duration, years**					0.351	0.839
1–5	121	60.50	132	57.90		
6–10	58	29.00	69	30.26		
>10	21	10.50	27	11.84		
**Hoehn-Yahr phase**					0.970	0.809
I	43	21.50	57	25.00		
II	79	39.50	86	37.72		
III	63	31.50	66	28.95		
IV	15	7.50	19	8.33		
**PD motor classification**					1.884	0.390
TD	57	28.50	79	34.65		
PIGD	41	20.50	44	19.30		
MXT	102	51.00	105	46.05		
**Family history of PD**	17	8.50	21	9.21	0.066	0.797
**Use of sleep medicines**	21	10.50	27	11.84	0.193	0.661
**Use of beta blockers**	28	14.00	33	14.47	0.020	0.889
**Use of ACEI**	12	6.00	21	9.21	1.543	0.214
**Use of calcium channel blockers**	14	7.00	17	7.46	0.033	0.856
**Symptoms severity on MDS-UPDRS-III**					0.555	0.758
Mild (0 ~ 32 score)	123	61.50	147	64.47		
Moderate (33 ~ 58 score)	69	34.50	71	31.14		
Severe (≥59 score)	8	4.00	10	4.39		
**Levodopa equivalent dose, mg**					0.166	0.920
<150	89	44.50	97	42.55		
150–300	77	38.50	91	39.91		
>300	34	17.00	40	17.54		

TD, tremor dominant; PIGD, postural instability/gait disorder; MXT, mixed type; ACEI, angiotensin-converting enzyme inhibitors; MDS-UPDRS-III, The Movement Disorder Society Unified Parkinson’s Disease Rating Scale-part III.

### 3.2 Content validity

The mean importance assigned to each item exceeded 4.0. The S-CVI was measured at 0.956, and the I-CVI ranged between 0.800 and 1.000, and all items were retained.

### 3.3 Item analysis

The CR values of all items were more than 3, no item need to be deleted. According to the criterion of correlation coefficient method, the coefficient value of item B4 was 0.365, despite the score was significantly correlated with the total scale (*p* < 0.01); the item-total coefficient values of other items ranged from 0.527 to 0.862. The Cronbach’s α coefficient was increased greatly if item B1, B4, and B5 was deleted, respectively (Table 2). After discussion with research groups, B1, B4, and B5 were deleted finally, and 19 items were retained.

**Table 2:** Item analysis of the FRPS-PD (*n* = 200).

Item	Critical ratio	Item-total correlation	Cronbach’s α if the item deleted	Note
A1	33.884^**^	0.862^**^	0.960	Retained
A2	23.787^**^	0.838^**^	0.960	Retained
A3	26.274^**^	0.823^**^	0.961	Retained
A4	30.160^**^	0.852^**^	0.960	Retained
A5	31.830^**^	0.860^**^	0.960	Retained
A6	22.094^**^	0.854^**^	0.960	Retained
A7	22.994^**^	0.822^**^	0.960	Retained
A8	18.277^**^	0.770^**^	0.961	Retained
A9	23.944^**^	0.821^**^	0.960	Retained
A10	16.641^**^	0.793^**^	0.961	Retained
A11	20.957^**^	0.815^**^	0.961	Retained
A12	16.803^**^	0.813^**^	0.961	Retained
A13	22.555^**^	0.836^**^	0.960	Retained
B1	5.460^**^	0.527^**^	0.964	Deleted
B2	16.340^**^	0.777^**^	0.961	Retained
B3	11.369^**^	0.662^**^	0.962	Retained
B4	4.949^**^	0.365^**^	0.965	Deleted
B5	9.156^**^	0.599^**^	0.964	Deleted
B6	8.747^**^	0.556^**^	0.963	Retained
B7	11.669^**^	0.662^**^	0.963	Retained
B8	14.379^**^	0.694^**^	0.962	Retained
B9	10.818^**^	0.577^**^	0.963	Retained

**p < 0.01.

### 3.4 Structure validity

**Figure 2:**
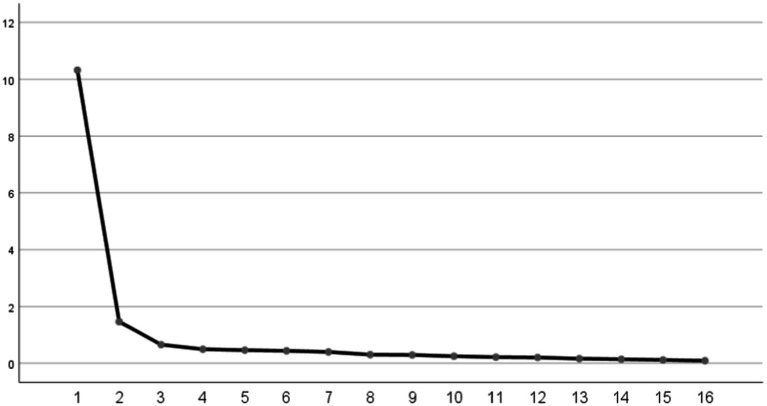
The scree plot.

Then EFA (*n* = 200) was performed. The initial EFA yielded a KMO value of 0.957 and a Bartlett’s sphericity test value of 3708.723 (*p* < 0.001), which demonstrated that the data set was adequate for EFA. In the first EFA, two common factors were extracted from 19 items in the scale. The cumulative contribution rate of common factors accounted to 71.19% (>60%). The item A9, B2, and B3 were removed because of the loads exceeding 0.4 on both common factors concurrently, as shown in Supplementary Tables S4, S5. In the second EFA, two common factors were extracted of 16 items with cumulative contribution rate of 73.67% (Tables 3, 4; Figure 2). Ultimately, the factor structure matched the predetermined dimensions, designating factor 1 as “risk perception” and factor 2 as “self-efficacy.”

**Table 3:** Analysis of the second EFA (*n* = 200).

Factor number	Initial eigenvalue			Extract the sum of squared loads			Rotating load sum of squares		
	Aggregate	Variance percentage	Accumulate %	Aggregate	Variance percentage	Accumulate %	Aggregate	Variance percentage	Accumulate %
1	10.321	64.505	64.505	10.321	64.505	64.505	8.402	52.513	52.513
2	1.466	9.164	73.669	1.466	9.164	73.669	3.385	21.156	73.669
3	0.656	4.099	77.768						
4	0.494	3.090	80.858						

**Table 4:** Factor loading of the second EFA (*n* = 200).

Item	Factor 1	Factor 2	Communality
A1 I’m at risk of falling when I start standing after sitting for a long time	**0.857**	0.343	0.852
A2 I’m at risk of falling when I start walking after standing for a long time	**0.819**	0.326	0.776
A3 I’m at risk of falling when I was standing and my limbs were shaking	**0.817**	0.239	0.724
A4 I’m at risk of falling when I make a turn	**0.853**	0.307	0.821
A5 I’m at risk of falling when I stop suddenly while walking	**0.855**	0.320	0.833
A6 I’m at risk of falling when I picked up speed suddenly	**0.844**	0.289	0.795
A7 I’m at risk of falling when I go up the stairs	**0.794**	0.314	0.729
A8 I’m at risk of falling when I go down the stairs	**0.797**	0.244	0.696
A10 I’m at risk of falling when I take things above the top of the head	**0.817**	0.203	0.709
A11 I’m at risk of falling when I get out of bed too fast	**0.822**	0.258	0.743
A12 I’m at risk of falling when I have pain in my feet and legs	**0.791**	0.273	0.701
A13 I’m at risk of falling when my feet and leg muscle have stiffness	**0.799**	0.323	0.744
B6 I can implement fall prevention according to the medical staff’s recommendation	0.259	**0.725**	0.592
B7 It is necessary for me to learn about the prevention of Parkinson’s disease	0.252	**0.789**	0.687
B8 It is necessary for me to do Parkinson’s rehabilitation to prevent falls	0.323	**0.790**	0.729
B9 I can call for help immediately after the fall	0.225	**0.779**	0.657

Varimax was the applied rotation method. Factor loadings with absolute values higher than 0.5 are in bold. Factor 1: Risk perception, Factor 2: Perceived benefits.

**Figure 3:**
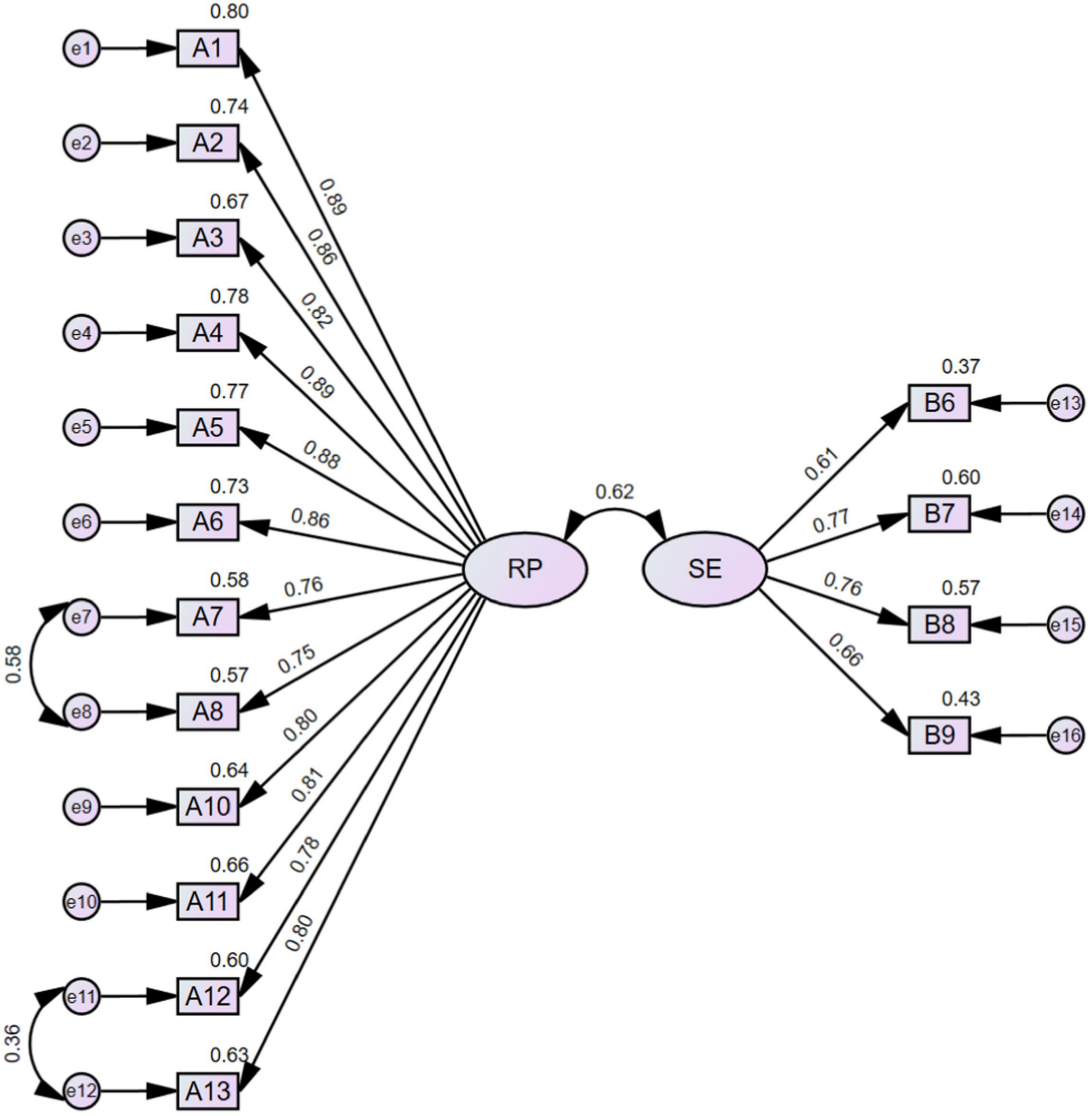
Structural model of FRPS-PD. RP, risk perception; SE, self-efficacy.

CFA (*n* = 228) was conducted to verify the rationality of the EFA-derived 16-item 2-factor structure of FRPS-PD. The initial model indices showed χ^2^/df = 3.293, RMSEA = 0.101, NFI = 0.892, TLI = 0.909, CFI = 0.922, GFI = 0.841, AGFI = 0.790, which demonstrated an inadequate fit according to the criterion (Table 5). Then, two paths of covariance between errors were added (e7 and e8, e11 and e12) based on the modification indices. The modified model achieved an acceptable fit to the data (χ^2^/df = 2.226, RMSEA = 0.074, NFI = 0.928, TLI = 0.951, CFI = 0.959, GFI = 0.887, AGFI = 0.848) (Table 5; Figure 3). The final Chinese version FRPS-PD was shown in Supplementary Table S6.

**Table 5:** Fitting results of CFA model of FRPS-PD (*n* = 228).

Commonly used standards	χ^2^/df	RMSEA	NFI	TLI	CFI	GFI	AGFI
Judgment criteria: good	<3	<0.050	>0.900	>0.900	>0.900	>0.900	>0.900
Judgment criteria: acceptable	<5	<0.080	>0.800	>0.800	>0.800	>0.800	>0.800
Results of initial model fitting	3.293	**0.101**	0.892	0.909	0.922	0.841	**0.790**
Result of modified model fitting	2.226	0.074	0.928	0.951	0.959	0.887	0.848

Values out of criterion are in bold.

### 3.5 Convergent and discriminant validity

The AVE value of risk perception dimension and self-efficacy dimension were 0.681 and 0.494, respectively, and the value of self-efficacy dimension was very close to 0.50. These indicated that acceptable convergent validity. In addition, the square root of AVE of risk perception dimension and self-efficacy dimension were 0.825 and 0.703, respectively. Both values exceeded each of their correlational coefficient of 0.618 between risk perception and self-efficacy, indicating robust good discriminative validity (Table 6).

**Table 6:** The results of convergent and discriminant validity analysis.

FRPS-PD	AVE	The square root of AVE	Inter-dimension correlations (*r*)	
			Risk perception	Self-efficacy
Risk perception	0.681	0.825	1.000	
Self-efficacy	0.494	0.703	0.484**	1.000

FRPS-PD, fall risk perception scale for PD patients; AVE, average variance extracted; r, Spearman’s rank correlation coefficient. **p < 0.01.

### 3.6 Reliability analysis

The Cronbach’s α coefficient of FRPS-PD was 0.952, and the Cronbach’s α coefficient of risk perception dimension and self-efficacy dimension were 0.963 and 0.781, respectively. The retest reliability coefficient (ICC) of the whole scale was 0.944, and the ICC of the risk perception dimension and the self-efficacy dimension were 0.940 and 0.912, respectively.

### 3.7 Floor/ceiling effect

The item of FRPS-PD score ranged from 1 to 5. The ceiling effect of FRPS-PD was highlighted at a significant level of 17.03% of all respondents. In contrast, no floor effect was detected (1.18%).

### 3.8 Criterion validity

Regarding the predictive validity, the FRPS-PD had significant positive correlation with MFS score (*r* = 0.567, *p* < 0.001).

## 4 Discussion

The aim of this study was to develop and validate a fall risk perception scale specifically for patients with Parkinson’s disease, named as FRPS-PD. Falls, particularly those that occur repeatedly, are of great concern in the management of PD (Pallavi et al., 2022), both for healthcare professionals and individuals with PD. The ability to accurately perceive the risk of falling is crucial for increasing awareness and implementing effective strategies to address these health challenges (Aycock et al., 2019). Therefore, it is imperative to comprehend the extent to which Parkinson’s patients perceive the risk of falling. To the best of our knowledge, this study is the first to develop a measurement tool for evaluating the level of fall risk perception among individuals with Parkinson’s disease. The FRPS-PD comprises two dimensions, encompassing a total of 16 items, with 12 items dedicated to risk perception and 4 items addressing self-efficacy. These items were derived from a comprehensive review of existing literature, as well as semi-structured interviews. Subsequently, the items were refined and adjusted through consultation with experts using the Delphi method, and further refined through pilot testing with people with PD. In addition, the psychological properties evaluation of the scale was conducted according to the guidelines of the COSMIN checklist (Prinsen et al., 2018; Terwee et al., 2018) to ensure the robustness of FRPS-PD. The results indicated satisfactory reliability and acceptable validity, suggesting a valid and reliable measurement for evaluating fall risk perception level for individuals with PD in mainland China.

The theoretical foundation of this scale is rooted in the Proactive Health theory and the RPA framework. Proactive Health theory emphasizes the individual’s perception of their own health, fostering positive behavioral shifts as a result of perception (Song et al., 2023; Wang et al., 2023). Given their medical condition, PD patients are more prone to falls and must remain vigilant (Van Bladel et al., 2023). However, continuous monitoring of patients with Parkinson’s disease by caregivers and medical staff is not feasible. Relying solely on passive fall prevention techniques, which rely on external monitoring, does not address the underlying cause of the high incidence of falls. The most effective approach involves increasing awareness among PD patients about their own safety aligning with the principle of Proactive Health. By being more aware, PD patients can actively mitigate potential hazards, leading to increased effectiveness with less effort (Christiansen et al., 2020). The RPA framework emphasizes the effects of risk perception and self-efficacy in shaping health protection behaviors (Rimal, 2003; Rimal et al., 2009). Based on the RPA framework, PD patients’ self-perceived fall risk and self-efficacy affect their willingness to prevent falling. Thus, the original hypothesized two domains were risk perception and self-efficacy, which had been verified by EFA analysis results.

The items were pooled through literature research and semi-structure interviews to ensure the rationality of the scale. The readability of the items was tested by pilot testing, and items with poor discriminative ability were identified and subsequently eliminated (Yamaguchi et al., 2022). In the initial phase of EFA, item A9, B2 and B3 exhibited factor loadings greater than 0.4 on both two common factors. As these items failed to discriminate between the dimensions they were intended to measure, they were subsequently removed. The second phase of EFA also extracted two common factors, which was adequate reflected the dimensionality of the structure of the theoretical framework on which it is based, named “risk perception” and “self-efficacy.” Subsequently, the structure underwent verification through CFA. The initial model’s fit indices demonstrated less satisfactory performance, prompting the incorporation of two error covariances as the modification recommendations (Figure 3). Eventually, all the model’s goodness-of-fit indices attained an acceptable level. Furthermore, the two-factor model exhibited good convergent and discriminant validity, further supporting its reasonable fit. In summary, the FRPS-PD demonstrates adequacy in measuring the fall risk perception level among individuals with PD.

Furthermore, the total Cronbach’s α coefficient of FRPS-PD demonstrated a high level of internal consistency, with a value of 0.952, indicating that all items effectively contribute to the measurement of the construct as a whole. Both factors (risk perception and self-efficacy) also exhibited good reliability, as their coefficients exceeded the threshold of 0.70. However, it is worth noting that the Cronbach’s α coefficient is influenced by the number of items, resulting in a lower value for the self-efficacy dimension (0.781, four items) compared to the risk perception dimension (0.963, 12 items). Additionally, the ICC score of FRPS-PD also demonstrated satisfactory stability for the scale. Nevertheless, we did not observe a floor effect, but rather a significant ceiling effect (17.03%). Generally, it is agreed that FRPS-PD is prone to a little bit ceiling effect due to its purpose of measuring fall risk perception in a specific patient population. This is because individuals with PD typically exhibit additional cardinal motor features (rigidity or rest tremor) (Poewe et al., 2017) that may serve as constant reminders of the risk of falling. Furthermore, regardless of whether individuals with PD are receiving outpatient or inpatient care, healthcare professionals will prioritize the identification and prevention of falls in order to promote heightened awareness of motor function and physical activity, thereby minimizing the occurrence of falls. The risk perception dimension of the FRPS-PD assesses the level of perceived fall risk with various activity status in people with PD. Consequently, the extent of the ceiling effect observed in this measurement is deemed acceptable. In the following phase, the research will focus on enlarging the sample size to thoroughly investigate the underlying causes and enduring impacts of the ceiling effect.

The concurrent validity of the FRPS-PD was not assessed due to the absence of a suitable gold standard instrument for evaluating fall risk perception. The Morse Fall Scale (MFS) is a widely recognized tool for measuring fall risk in adults, both domestically and internationally (Morse et al., 1989; Kim et al., 2022). Thus, we tested the predictive validity based on the correlations between scores of FRPS-PD and MFS as a nap of criterion validity. The results revealed moderately positive significant correlations, suggesting that PD patients with higher level of fall risk perception are more likely to experience actual falls. The recently updated World Guidelines for Fall Prevention and Management strongly recommend that the incorporation of patients’ apprehensions regarding falls within a comprehensive multi-factor assessment (Montero-Odasso et al., 2022). The FRPS-PD has the potential to encourage patients with PD to adopt fall prevention measures, foster proactive protection (Solares et al., 2023), and improve quality of life (Sangarapillai et al., 2021). Additionally, it can aid healthcare professionals in identifying individuals with biased perceptions and promptly implementing personalized interventions to improve the efficacy of fall prevention strategies and reduce the occurrence of adverse events.

Several limitations should be acknowledged. First, although we tried to control the biases of the results by expanding the sample from three hospitals in different cities, the generalizability of the results of this study might still be threatened, as the enrolled participants were absence from obvious cognition dysfunction, and more than half of the individuals had mild symptom severity based on MDS-UPDRS-III and PD duration with less than 5 years. Future research should include more diverse samples covering more PD patients with moderate or above symptoms severity to guarantee adequate generalizability of this scale, since whose patients had higher fall risk than patients with mild symptoms. Second, the sample of CFA was also enrolled from same hospitals with EFA, thus it is difficult to report the feasibility of FRPS-PD in other hospitals or communities or in other countries. As such, future studies need to test the scale in different levels of hospitals or communities. Third, the calculation of the cut-off value of FRPS-PD for varying levels of risk perception was not feasible due to the limited samples and the characteristics of disease. It is important to note that patients’ motor symptoms may undergo alterations as a result of disease progression or modifications in treatment, thereby significantly impacting their perception of fall risk. Consequently, future research endeavors should aim to augment the sample size and categorize patients into distinct subgroups based on the severity of PD in order to investigate the appropriate cut-off value for risk perception in accordance with their specific disease characteristics.

## 5 Conclusion

The 16-item, 2-dimension FRPS-PD has satisfactory reliability and acceptable validity. It is an effective instrument for evaluating fall risk perception level for PD patients in mainland China and consequently helping healthcare professionals to identify individuals with misperception toward fall risk, then implement targeted interventions. Nevertheless, its application warrants further investigation in larger samples of PD in different settings and areas.

## Data availability statement

The raw data supporting the conclusions of this article will be made available by the authors, without undue reservation.

## Ethics statement

This study was reviewed and approved by the Second Affiliated Hospital of Zhejiang University School of Medicine. All subjects agreed to participate in the study voluntarily.

## Author contributions

XY: Conceptualization, Data curation, Formal analysis, Methodology, Software, Writing – original draft, Investigation, Visualization, Writing – review & editing. MY: Conceptualization, Methodology, Supervision, Validation, Writing – review & editing, Funding acquisition, Project administration. ZG: Supervision, Writing – review & editing, Formal analysis. XS: Supervision, Writing – review & editing, Resources. JJ: Project administration, Supervision, Writing – review & editing, Funding acquisition, Validation.
