# Supplementary material for: Development and validation of fall risk perception scale for patients with Parkinson’s disease
Source: Front Psychol. 2024 Feb 28;15:1289067. doi: 10.3389/fpsyg.2024.1289067 (PMC10932967; doi:10.3389/fpsyg.2024.1289067)
Supplement: Supplementary file 1 [file Data_Sheet_1.doc]

**Supplementary Files**

**Table S1 The outline of semi-structured interviews**

| No. | Question |
| --- | --- |
| 1 | Under what circumstances do you think you are prone to falls? |
| 2 | Do you think you are at high risk of falling？ |
| 3 | Do you worry about falling？ |
| 4 | How do you keep from falling？ |

**Table S2 The original version of FRPS-PD**

| No. | Item content |
| --- | --- |
| A1 | I'm at risk of falling when I have trouble getting started |
| A2 | I'm at risk of falling when I have hallucinations |
| A3 | I'm at risk of falling when my limbs were shaking |
| A4 | I'm at risk of falling when the strength of my feet was weak |
| A5 | I'm at risk of falling when I have pain in my feet and legs |
| A6 | I'm at risk of falling when my feet and leg muscle have stiffness |
| A7 | I'm at risk of falling when my leg muscle spasms |
| A8 | I'm at risk of falling when I start walking after sitting for a long time |
| A9 | I'm at risk of falling when I start walking after standing for a long time |
| A10 | I'm at risk of falling when I make a turn |
| A11 | I'm at risk of falling when I go up the stairs |
| A12 | I'm at risk of falling when I go down the stairs |
| A13 | I'm at risk of falling when I go over obstacles |
| A14 | I'm at risk of falling when I picked up speed suddenly |
| A15 | I'm at risk of falling when I take things above the top of the head |
| A16 | I'm at risk of falling when I stand to wear or take off their shoes |
| A17 | I'm at risk of falling when I stand on the bus |
| B1 | I worried extremely about falling may paradoxically increase the risk of falling |
| B2 | I know how to protect myself when I experience a fall |
| B3 | I want to do some exercise to prevent falling |
| B4 | I want to learn something about falling |
| B5 | I want to know about the side effects of Parkinson's drugs |
| B6 | I am willing to use a walker to prevent falling if required |
| B7 | I will implement fall prevention according to the nurses’ recommendation |
| B8 | I will wear non-slip shoes when I am going out |
| B9 | I will try to keep myself from falling when I'm hallucinating |
| B10 | I won’t go out alone with tremors |
| B11 | I will avoid sitting or standing for a long time |
| B12 | I will call for help immediately after falling |

**Table S3 The pilot testing version of** **FRPS-PD**

| No. | Original No. | Item |
| --- | --- | --- |
| A1 | A8 | I'm at risk of falling when I start standing after sitting for a long time |
| A2 | A9 | I'm at risk of falling when I start walking after standing for a long time |
| A3 | A3 | I'm at risk of falling when I were standing and my limbs were shaking |
| A4 | A10 | I'm at risk of falling when I make a turn |
| A5 | added | I'm at risk of falling when I stop suddenly while walking |
| A6 | A14 | I'm at risk of falling when I picked up speed suddenly |
| A7 | A11 | I'm at risk of falling when I go up the stairs |
| A8 | A12 | I'm at risk of falling when I go down the stairs |
| A9 | A13 | I'm at risk of falling when I go over obstacles |
| A10 | A15 | I'm at risk of falling when I take things above the top of the head |
| A11 | added | I'm at risk of falling when I get out of bed too fast |
| A12 | A5 | I'm at risk of falling when I have pain in my feet and legs |
| A13 | A6 | I'm at risk of falling when my feet and leg muscle have stiffness |
| B1 | B11 | I can avoid sitting or standing for a long time |
| B2 | B10 | I can prevent falling when my limbs shake |
| B3 | A2 and B9 | I will try to keep myself from falling when I'm hallucinating |
| B4 | B2 | I can protect myself when I experience a fall |
| B5 | B6 | I am willing to use a walker to prevent falling if required |
| B6 | B7 | I can implement fall prevention according to the medical staff's recommendation |
| B7 | B4 | It is necessary for me to learn about the prevention of Parkinson's disease |
| B8 | B3 | It is necessary for me to do Parkinson's rehabilitation to prevent falls |
| B9 | B12 | I can call for help immediately after the fall |

**Table S4 The results of first EFA**

| Factor number | Initial eigenvalue | | | Extract the sum of squared loads | | | Rotating load sum of squares | | |
| --- | --- | --- | --- | --- | --- | --- | --- | --- | --- |
|  | [aggregate](javascript:;) | variance percentage | accumulate % | [aggregate](javascript:;) | variance percentage | accumulate % | [aggregate](javascript:;) | variance percentage | accumulate % |
| 1 | 12.016 | 63.242 | 63.242 | 12.016 | 63.242 | 63.242 | 9.372 | 49.328 | 49.328 |
| 2 | 1.511 | 7.953 | 71.194 | 1.511 | 7.953 | 71.194 | 4.155 | 21.866 | 71.194 |
| 3 | 0.792 | 4.168 | 75.363 |  |  |  |  |  |  |
| 4 | 0.636 | 3.348 | 78.710 |  |  |  |  |  |  |
| 5 | 0.503 | 2.646 | 81.357 |  |  |  |  |  |  |
| 6 | 0.459 | 2.414 | 83.770 |  |  |  |  |  |  |
| 7 | 0.438 | 2.305 | 86.076 |  |  |  |  |  |  |
| 8 | 0.384 | 2.021 | 88.096 |  |  |  |  |  |  |
| 9 | 0.327 | 1.721 | 89.817 |  |  |  |  |  |  |
| 10 | 0.314 | 1.652 | 91.470 |  |  |  |  |  |  |
| 11 | 0.279 | 1.469 | 92.939 |  |  |  |  |  |  |
| 12 | 0.263 | 1.384 | 94.322 |  |  |  |  |  |  |
| 13 | 0.216 | 1.139 | 95.462 |  |  |  |  |  |  |
| 14 | 0.205 | 1.078 | 96.540 |  |  |  |  |  |  |
| 15 | 0.180 | 0.946 | 97.486 |  |  |  |  |  |  |
| 16 | 0.149 | 0.786 | 98.272 |  |  |  |  |  |  |
| 17 | 0.128 | 0.674 | 98.945 |  |  |  |  |  |  |
| 18 | 0.113 | 0.595 | 99.541 |  |  |  |  |  |  |
| 19 | 0.087 | 0.459 | 100.000 |  |  |  |  |  |  |

**Table S5 Factor load and common degree of the first EFA (*n*=200)**

| Item | Factor 1 | Factor 2 | communality |
| --- | --- | --- | --- |
| A1 I'm at risk of falling when I start standing after sitting for a long time | **0.852** | 0.342 | 0.843 |
| A2 I'm at risk of falling when I start walking after standing for a long time | **0.814** | 0.315 | 0.763 |
| A3 I'm at risk of falling when I was standing and my limbs were shaking | **0.806** | 0.272 | 0.724 |
| A4 I'm at risk of falling when I make a turn | **0.847** | 0.334 | 0.829 |
| A5 I'm at risk of falling when I stop suddenly while walking | **0.850** | 0.334 | 0.834 |
| A6 I'm at risk of falling when I picked up speed suddenly | **0.836** | 0.307 | 0.794 |
| A7 I'm at risk of falling when I go up the stairs | **0.791** | 0.329 | 0.733 |
| A8 I'm at risk of falling when I go down the stairs | **0.792** | 0.250 | 0.690 |
| A9 I'm at risk of falling when I go over obstacles | **0.739** | **0.431** | 0.733 |
| A10 I'm at risk of falling when I take things above the top of the head | **0.811** | 0.214 | 0.703 |
| A11 I'm at risk of falling when I get out of bed too fast | **0.820** | 0.248 | 0.734 |
| A12 I'm at risk of falling when I have pain in my feet and legs | **0.784** | 0.295 | 0.701 |
| A13 I'm at risk of falling when my feet and leg muscle have stiffness | **0.791** | 0.328 | 0.734 |
| B2 I can prevent falling when my limbs shake | **0.575** | **0.513** | 0.593 |
| B3 I will try to keep myself from falling when I'm hallucinating | **0.488** | **0.537** | 0.527 |
| B6 I can implement fall prevention according to the medical staff's recommendation | 0.245 | **0.716** | 0.573 |
| B7 It is necessary for me to learn about the prevention of Parkinson's disease | 0.234 | **0.810** | 0.710 |
| B8 It is necessary for me to do Parkinson's rehabilitation to prevent falls | 0.308 | **0.790** | 0.720 |
| B9 I can call for help immediately after the fall | 0.215 | **0.737** | 0.590 |

**Table S6 Chinese version of FRPS-PD**

| **条目** | **评分表** | | | | |
| --- | --- | --- | --- | --- | --- |
|  | **非常不同意** | **不同意** | **不确定** | **同意** | **非常同意** |
| A1.我久坐后站起会有跌倒风险 |  |  |  |  |  |
| A2.我久站后起步会有跌倒风险 |  |  |  |  |  |
| A3.我站立时肢体抖动会有跌倒风险 |  |  |  |  |  |
| A4.我步行转弯时会有跌倒风险 |  |  |  |  |  |
| A5.我行走时突然停止会有跌倒风险 |  |  |  |  |  |
| A6.我走路时突然前冲会有跌倒的风险 |  |  |  |  |  |
| A7.我上楼梯时会有跌倒风险 |  |  |  |  |  |
| A8.我下楼梯时会有跌倒风险 |  |  |  |  |  |
| A10.我踮脚取高处的物品时会有跌倒风险 |  |  |  |  |  |
| A11.我快速离床时会有跌倒风险 |  |  |  |  |  |
| A12.我腿脚剧烈疼痛时会有跌倒风险 |  |  |  |  |  |
| A13.我腿脚肌肉僵硬时会有跌倒风险 |  |  |  |  |  |
| B6.我能够遵守医务人员的嘱咐防范跌倒 |  |  |  |  |  |
| B7.我有必要学习帕金森病患者的防跌知识 |  |  |  |  |  |
| B8.我有必要进行帕金森病康复训练以预防跌倒 |  |  |  |  |  |
| B9.跌倒后我会立即呼救 |  |  |  |  |  |

注：A-风险感知维度；B-自我效能维度。
